# A Challenging Case: Unraveling the Complexities of Undifferentiated Connective Tissue Disease

**DOI:** 10.7759/cureus.62818

**Published:** 2024-06-21

**Authors:** Malay Rathod, Shivani Modi, Supriya Peshin, Alyssa Kang

**Affiliations:** 1 Medicine, Monmouth Medical Center, Long Branch, USA; 2 Internal Medicine, Jefferson Einstein Healthcare Network, Norristown, USA; 3 Internal Medicine, Norton Community Hospital, Norton, USA

**Keywords:** hydroxychloroquine, treatment, diagnosis, autoimmune disorder, undifferentiated connective tissue disease

## Abstract

Undifferentiated connective tissue disease (UCTD) poses a significant diagnostic challenge due to its wide array of clinical presentations and the absence of specific diagnostic criteria. We present the case of a 22-year-old female who initially exhibited symptoms resembling the common flu, including recurrent fever, headaches, and constitutional symptoms. Despite initial tests showing no abnormalities and negative autoimmune serology, her symptoms persisted, prompting further investigation. Although subsequent imaging studies yielded no definitive diagnosis, her elevated inflammatory markers and progressively positive antinuclear antibody (ANA) test after the initial negative result suggested an underlying autoimmune process.

After six months of persistent symptoms, treatment with hydroxychloroquine initiated by a rheumatologist led to a remarkable resolution of her symptoms. This case highlights the challenge of diagnosing UCTD, particularly in young individuals, where symptoms may manifest early in life without clear laboratory abnormalities, sometimes with initial negative ANA, making it a learning point to recheck ANA levels even if they were initially negative. It underscores the importance of considering UCTD in patients with unexplained symptoms and inconclusive laboratory findings, as early initiation of appropriate treatment can significantly alleviate symptoms and improve patient outcomes.

## Introduction

Connective tissue, essential for organ function, is a complex network providing support and cohesion among various tissues. However, when the immune system targets these tissues, it can lead to a wide spectrum of symptoms and conditions falling under the umbrella term of connective tissue diseases. Each specific disease within this category has its own diagnostic criteria. Yet, when a patient’s symptoms do not align with any established criteria, they may receive a diagnosis of undifferentiated connective tissue disease (UCTD) [1].

UCTD is characterized by both clinical symptoms and serological markers typical of systemic autoimmune disorders but does not fit neatly into any single category such as lupus, scleroderma, or rheumatoid arthritis. First identified by LeRoy et al. in 1980, UCTD represents an early stage of a major rheumatic disease [1,2].

The prevalence of UCTD is notable, with around 90% of cases observed in women in the United States, particularly between the ages of 32 and 44. Interestingly, most cases do not progress to complete connective tissue disorders. Instead, patients often exhibit moderate clinical symptoms and a negative autoantibody profile over time, remaining stable [1]. In the United States, roughly half of individuals with a connective tissue disorder have an underlying UCTD, highlighting the significance of its recognition. Moreover, the demographics of UCTD cases vary, with approximately 72% diagnosed in Caucasian populations. Globally, females represent up to 78% of cases, with even higher rates observed in certain regions such as Italy and Hungary [2].

Through the presentation of clinical cases, we aim to address the diagnostic challenges posed by UCTD, emphasizing the importance of early recognition and appropriate management. By enhancing awareness and understanding among healthcare providers, we strive to improve patient care and outcomes in this complex and often misunderstood condition.

## Case presentation

A 22-year-old white female with a history of anxiety and depression initially presented to the clinic with symptoms of low-grade fever, runny nose, congestion, cough, and headaches for the last 7-10 days. She had not seen Psychiatry since the age of 18 years and a psychiatry referral was provided. Despite testing negative for flu, her fever persisted, accompanied by daily headaches, which were relieved with acetaminophen 650 mg daily. She denied any travel history or tick bites. Initial laboratory tests, including complete blood count (CBC), complete metabolic panel (CMP), thyroid-stimulating hormone, tuberculosis blood test, sexually transmitted infection test, urinalysis (UA), and chest X-ray, were within normal limits except for a slightly elevated white blood cell (WBC) count of 12.3 and C-reactive protein (CRP) of 13. She was advised to monitor her temperature alongside her menstrual cycle. At this point, the patient was recommended to follow up with gynecology.

Upon follow-up (five weeks later), the patient’s symptoms persisted, with fever ranging from 99°F to 102°F and more frequent headaches requiring increased use (acetaminophen 650 mg twice daily). At this moment, the patient was recommended to follow up with psychiatry to rule out any psychiatric conditions. The patient failed to follow up with gynecology and was recommended to do so. Imaging studies which included CT scans of the head and chest/abdomen/pelvis were unremarkable. Repeat laboratory tests showed a WBC count of 11.2, absolute neutrophil count of 7.7, CRP of 43, and erythrocyte sedimentation rate of 26. UA revealed leukocytes and bacteria, with a positive urine culture despite the absence of urinary symptoms. Human immunodeficiency virus and hepatitis tests were negative.

On the next follow-up visit (five weeks later, a total of 10 weeks after presentation), a repeat UA was obtained which was negative. The patient stated that her fevers persisted, and she required acetaminophen 650 mg twice daily as before. She complained of feeling more tired but other than that symptoms had not changed. Her headache now occurred every day which was relieved by Tylenol. We suggested she reduce her usage of Tylenol as this could be a refractory headache. A neurology referral was provided. MRI was ordered. A repeat antinuclear antibody (ANA) was ordered at this visit. The patient was advised to follow up with psychiatry and gynecology.

**Figure 1:**
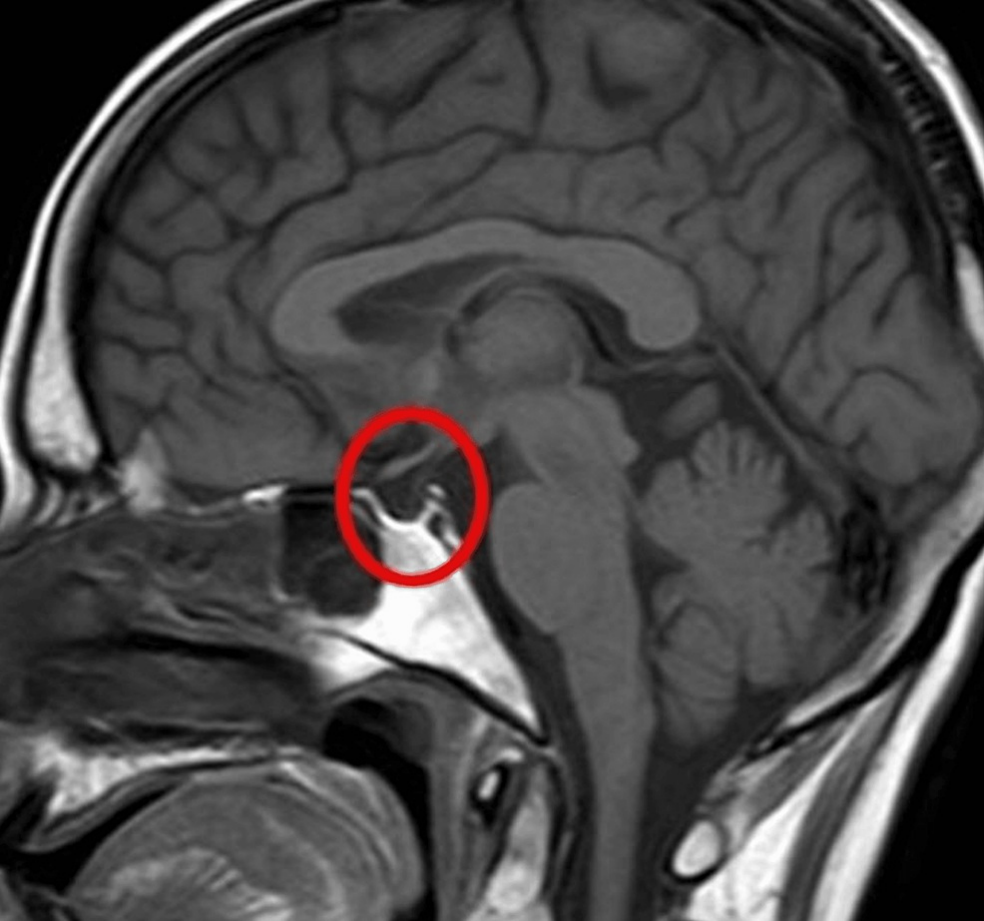
Brain MRI with the circle showing mild empty sella turcica.

Further investigations, including blood smear, malaria test, autoimmune markers (ANA, anti-dsDNA, anti-Smith, anti-U1 RNP antibodies, rheumatoid factor), and repeat CBC and CMP, were negative except for an elevated ANA titer of 1:640 and C3 complement of 178. Due to an increase in headache frequency and obesity in a young female, there was concern about pseudotumor cerebri, and an MRI was ordered. MRI of the brain revealed a mild empty sella turcica, as shown in Figure 1. Neurology started the patient on a Sumatriptan trial to confirm the diagnosis of migraine. The patient stated that her headache resolved after taking Sumatriptan for a few days. Referrals to rheumatology were made.

Rheumatology initiated further testing along with a repeat autoimmune serology workup, revealing an ANA titer of 1:1,280. A trial of hydroxychloroquine 200 mg twice daily for four to six months was initiated, resulting in the resolution of all symptoms. The patient was clinically diagnosed with unspecified systemic connective tissue disease. Currently, she is following up with a rheumatologist as per schedule.

## Discussion

When the immune system attacks our connective tissue, UTDC occurs. UCTD is diagnosed using a variety of definitions and standards. Exposure to certain environmental conditions, such as pollution and cigarette smoke, may initiate it. The clinical manifestations of a systemic autoimmune disease combined with laboratory proof of autoimmunity, together with the patient’s non-fulfillment of any commonly used classification criteria for traditional autoimmune diseases, are the hallmarks of UCTD. It is a diagnosis of exclusion [1]. In this case, the diagnosis of UCTD was confirmed approximately six months after the initial encounter, after ruling out more common etiologies during the diagnostic process.

UCTD is classified into two types based on how it progresses toward a recognizable autoimmune disease. The two subtypes are as follows: emerging (eUCTD) and stable (sUCTD). eUCTD is a more aggressive form and progresses within a few years into another autoimmune disease such as rheumatoid arthritis or systemic lupus erythematosus (SLE). sUCTD is stable and remains dormant for years without any progression and has better outcomes. The most common one is eUCTD seen in up to 28% of patients which further progresses to SLE or rheumatoid arthritis within five to six years [2]. In our case, the patient presented at the age of 22, which is much earlier than expected for the average age population.

In 1999, preliminary criteria were proposed to diagnose UTCD. The following criteria must be met: a clinical presentation suggestive of a specific connective tissue disease but does not meet its criteria [2]; two separate positive serological markers, including a positive ANA marker [3]; and symptoms that have persisted for at least three years [4].

Common symptoms of the disease are arthralgia in up to 86% of the patients and various skin lesions including livedo, purpura, acrocyanosis, and telangiectasias. Other common symptoms include the Raynaud phenomenon (33%), sicca symptoms (30%), mucocutaneous symptoms, such as oral ulcers (23%), arthritis (22%), fever (15%), and thyroid disease (7%). For certain patients, the initial presentation may consist of constitutional symptoms such as fever, fatigue, and feeling unwell [3]. In our case, the patient presented with symptoms of seasonal flu but no improvement in fever and persistently elevated WBC led to further investigation of this case with differential diagnosis including the spectrum of rheumatological disease. Although anti-Ro/SSA and anti-U1-RNP are thought to be the most commonly found markers in this disease, ANA is a highly prevalent immunological profile in this population.

UTCD has a milder disease course. Usually, there is no involvement of severe organ damage, especially renal and nervous systems. The survival rate in UCTD at 10 years is 91%. It is primarily managed as an outpatient. Hydroxychloroquine is the most commonly prescribed medication in patients presenting with arthritis. A short course of low-dose glucocorticoids is commonly prescribed in patients with arthritis and serositis treated with non-steroidal anti-inflammatory drugs but showing no improvement. Common immunosuppressants used include azathioprine, methotrexate, and mycophenolate mofetil [1,2]. Our patient improved with hydroxychloroquine which supports the diagnosis.

Patients usually feel uneasy, mostly due to the unpredictability of the disease course. Patients sometimes also feel that their concerns are not addressed well by the healthcare professional as the disease does not require intensive follow-up and lab testing. Emotional disturbances can impact their lives as well, so it is imperative to educate patients about the disease and its outcomes [4].

UCTD poses significant challenges in diagnosis, prognosis, and management due to its varied clinical presentation and uncertain progression. While up to 60% of cases may remain undifferentiated, about 10-20% of patients may experience symptom remission or subside without progressing to a defined connective tissue disease. However, for those who do progress, the risk is highest within the first five years of onset, highlighting the importance of close monitoring during this period [1].

Factors such as cytopenias, nail fold capillaroscopy changes, and ANA titers may offer insights into the likelihood of disease progression. Patients facing UCTD may experience impaired quality of life, both physically and mentally, necessitating careful management and support [1,2].

Complications vary depending on the affected organ systems, ranging from interstitial lung disease to cardiomegaly. While severe organ involvement and life-threatening conditions are possible, the evolution of stable UCTD into a defined disease, such as SLE, occurs in a minority of cases, particularly if the condition remains stable for more than three years [1].

Pregnant individuals with UCTD require close clinical observation, as they may experience disease exacerbation or progression during pregnancy. Early recognition and patient education are essential for effective management and symptom control. Collaboration between primary care providers and specialists, particularly rheumatologists, is crucial for optimizing patient outcomes and promoting overall well-being [1].

## Conclusions

Despite ongoing uncertainties surrounding UCTD, continued research for early diagnosis and clinical guidelines are needed to enhance understanding and improve patient care. Early recognition and raising awareness about UCTD are crucial for empowering patients to effectively manage and control their condition. Patient education regarding typical triggers and offending substances, as well as symptoms warranting immediate medical attention, can aid in symptom management, shorten illness duration, and prevent complications.

UCTD stems from diverse causes and can impact multiple organs based on individual susceptibility. Thus, fostering collaboration between primary care providers and specialists, particularly rheumatologists, is essential for achieving optimal patient outcomes. Acknowledging patient’s emotions and making them feel heard can go a long way along with medications. This coordinated approach ensures comprehensive care tailored to the patient’s unique needs, promoting better disease management and overall well-being.
